# Bibliometric analysis of residual cardiovascular risk: trends and frontiers

**DOI:** 10.1186/s41043-023-00478-z

**Published:** 2023-11-28

**Authors:** Lin Wang, Sutong Wang, Chaoyuan Song, Yiding Yu, Yuehua Jiang, Yongcheng Wang, Xiao Li

**Affiliations:** 1 First Clinical Medical College, Shandong University of Traditional Chinese Medicine, Jinan, China; 2 Department of Cardiovascular, Affiliated Hospital of Shandong University of Traditional Chinese Medicine, Jinan, China; 3 Central Laboratory, Affiliated Hospital of Shandong University of Traditional Chinese Medicine, Jinan, China; 4 Department of Neurology, Zibo Central Hospital, Zibo, China

**Keywords:** Residual cardiovascular risk, Bibliometric analysis, CiteSpace, VOSviewer

## Abstract

**Background:**

The presence of residual cardiovascular risk is an important cause of cardiovascular events. Despite the significant advances in our understanding of residual cardiovascular risk, a comprehensive analysis through bibliometrics has not been performed to date. Our objective is to conduct bibliometric studies to analyze and visualize the current research hotspots and trends related to residual cardiovascular risk. This will aid in understanding the future directions of both basic and clinical research in this area.

**Methods:**

The literature was obtained from the Web of Science Core Collection database. The literature search date was September 28, 2022. Bibliometric indicators were analyzed using CiteSpace, VOSviewer, Bibliometrix (an R package), and Microsoft Excel.

**Result:**

A total of 1167 papers were included, and the number of publications is increasing rapidly in recent years. The United States and Harvard Medical School are the leading country and institution, respectively, in the study of residual cardiovascular risk. Ridker PM and Boden WE are outstanding investigators in this field. According to our research results, the New England Journal of Medicine is the most influential journal in the field of residual cardiovascular risk, whereas Atherosclerosis boasts the highest number of publications on this topic. Analysis of keywords and landmark literature identified current research hotspots including complications of residual cardiovascular risk, risk factors, and pharmacological prevention strategies.

**Conclusion:**

In recent times, global attention toward residual cardiovascular risk has significantly increased. Current research is focused on comprehensive lipid-lowering, residual inflammation risk, and dual-pathway inhibition strategies. Future efforts should emphasize strengthening international communication and cooperation to promote the comprehensive evaluation and management of residual cardiovascular risk.

**Supplementary Information:**

The online version contains supplementary material available at 10.1186/s41043-023-00478-z.

## Introduction

Cardiovascular disease (CVD) is the main cause of global health hazards, which brings a great burden on society. In 2016, approximately 31% of global deaths were attributed to CVD [1]. In recent years, the treatment of CVD has made unprecedented progress, resulting in decreased mortality rates in developed countries. These improvements are largely due to interventions targeting risk factors, such as lifestyle modifications, blood pressure and glycemic control, antiplatelet therapies, and high-dose statin treatments. However, even if standard evidence-based treatment is adopted, some patients still have residual cardiovascular risk [2].

Residual cardiovascular risk is defined as the persistent risk of cardiovascular events in patients whose major risk factors have been controlled through current evidence-based recommended treatments [3]. Lipid-lowering is fundamental in treating atherosclerosis and its complications; however, cardiovascular events still occur frequently even after lowering low-density lipoprotein cholesterol (LDL-C) to guideline-recommended levels [4]. The presence of residual cardiovascular risk suggests that sole reliance on the control of traditional risk factors is inadequate. This may be due to other mechanisms such as inflammation, platelet activation, and oxidative stress. In recent years, numerous studies have focused on how to reduce residual cardiovascular risk. For example, targeted anti-inflammatory drugs like canakinumab or low-dose colchicine have shown promise in clinical trials for reducing cardiovascular events [5–8]. Additionally, novel lipid-lowering agents such as Proprotein convertase subtilisin/kexin type 9 (PCSK9) inhibitors and inclisiran present new opportunities for further risk reduction [9–11]. Therefore, beyond actively controlling traditional risk factors, it is crucial to continuously explore and identify new cardiovascular risk factors and develop effective interventions to mitigate residual cardiovascular risk. It is important to note that despite the emergence of new technologies and medications, lifestyle modification remains the cornerstone of CVD prevention. Furthermore, the value of interdisciplinary collaboration is on the rise. Internists, nutritionists, rehabilitation therapists, and other experts must collaborate to provide comprehensive care for patients. With the advancement of digital medical technology, telemedicine, wearable devices, health apps, and other tools are becoming increasingly significant in the management of CVD [12]. These technologies not only allow doctors to assess and monitor patients' health status more conveniently but also empower patients to take a more active role in managing their health, thereby further diminishing residual cardiovascular risk.

The field in question has attracted a great deal of attention from researchers, resulting in a vast body of literature. A systematic review of this literature is invaluable for scholars who wish to gain a comprehensive overview of current research in the field and identify future research directions. Bibliometrics is an advanced method to understand the trajectory and frontiers of the discipline [13]. Bibliometrics allows quantitative analysis of the quantity, authors, journals, institutions, citations, and themes of academic literature, helping researchers to evaluate the quality, impact, and visibility of scholarly results [14, 15]. In addition, the interconnections between the different components of a study are revealed visually through bibliometric software, enabling scholars to build a comprehensive knowledge structure based on thematic clusters relevant to their research area [16].

To our knowledge, there is currently no bibliometric analysis available on residual cardiovascular risk. The aim of our study is to analyze the publications in this field using the WoSCC database and to clarify the current state and dynamic changes in research on residual cardiovascular risk. Importantly, it provides valuable insights for researchers to understand the hotspots and emerging trends in the field.

## Methods

### Data collection

**Fig. 1:**
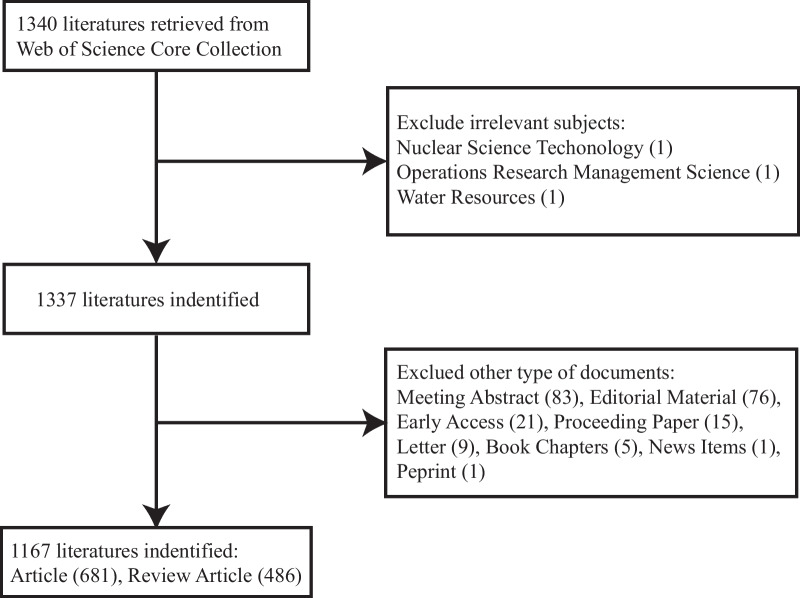
Flowchart of the inclusion and exclusion criteria

The literature was retrieved from the Web of Science Core Collection database (WoSCC), and the retrieval strategy was as follows: TS = ((cardiovascular OR cardiac OR heart* OR coronary OR atherosis OR atherosclerosis OR circulation OR CVD) AND ("residual risk*" OR "Residual ASCVD Risk*" OR "residual Cardiovascular risk*" OR "residual Coronary Risk*" OR "Residual cardiac risk*" OR "residual CVD risk*" OR "residual metabolic risk*" OR "Residual inflammation risk*" OR "Residual lipid risk*")). In our analysis, we have restricted the types of literature to reviews and original research studies. The time span is not limited, and the entire retrieval process was completed on September 28, 2022, at 21:26. A total of 1340 articles were retrieved. By eliminating irrelevant documents and duplicate documents, 1167 documents were finally obtained, which is shown in Fig. 1.

### Analysis method and tools

In our study, we first exported the search results as a plain text file and merged synonyms to enhance the accuracy of the subsequent bibliometric analysis. We then used a suite of advanced bibliometric tools, including VOSviewer (version 1.6.18), CiteSpace (version 6.1.R3), Microsoft Excel (2019), and the R bibliometrix package, to conduct a thorough analysis and visualization of the data. Specifically, we used CiteSpace to examine the literature pertinent to this study's topic and to identify the evolution paths and key turning points within the domain [17]. VOSviewer is a scientific knowledge mapping software that renowned for its powerful graphical display capabilities and its suitability for large-scale data analysis [18]. Bibliometrix is an analysis tool for scientometrics developed by Massimo Aria and Corrado Cuccurullo. As Bibliometrix is written in the R programming language, it provides a high level of flexibility and seamless integration with other statistical packages in the R ecosystem. This feature enables researchers to customize their bibliometric analysis by leveraging the extensive range of statistical tools and functions available in R [19]. We conducted a comprehensive bibliometric analysis using Microsoft Excel and Bibliometrix to calculate key metrics such as publication counts, total citations, and h-index values at the levels of author, journal, institution, and country. We also used Microsoft Excel to create visualizations of national publications and author citations. We performed co-occurrence analysis between countries, journals, and keywords using VOSviewer. Additionally, we used CiteSpace to analyze institutional co-occurrence, references co-citation patterns, and identify bursts in research theme.

## Results

### Analysis of annual publication volume

**Fig. 2:**
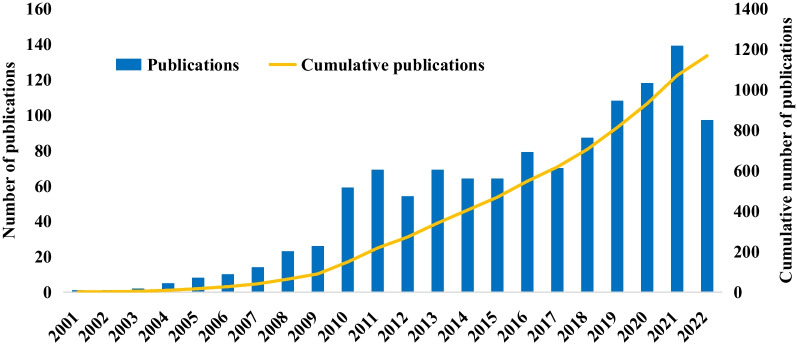
Changes in the number of papers on residual cardiovascular risk studies over time

The analysis of the collected literature reveals that studies on residual cardiovascular risk first emerged in 2001. We analyzed trends in publications from 2001 to the present, as shown in Fig. 2. Over the past 22 years, the annual volume of publications in this field has shown a consistent increase. This upward trajectory allows us to categorize the publication volume into three distinct phases. The first phase was from 2001 to 2009, with a small number of articles and slow growth, which was the beginning of residual cardiovascular risk studies. In the second stage, from 2010 to 2017, the number of papers fluctuated steadily. In the third phase, from 2018 to the present, the number of publications has continued to grow rapidly.

### Contributions of countries/regions

**Fig. 3:**
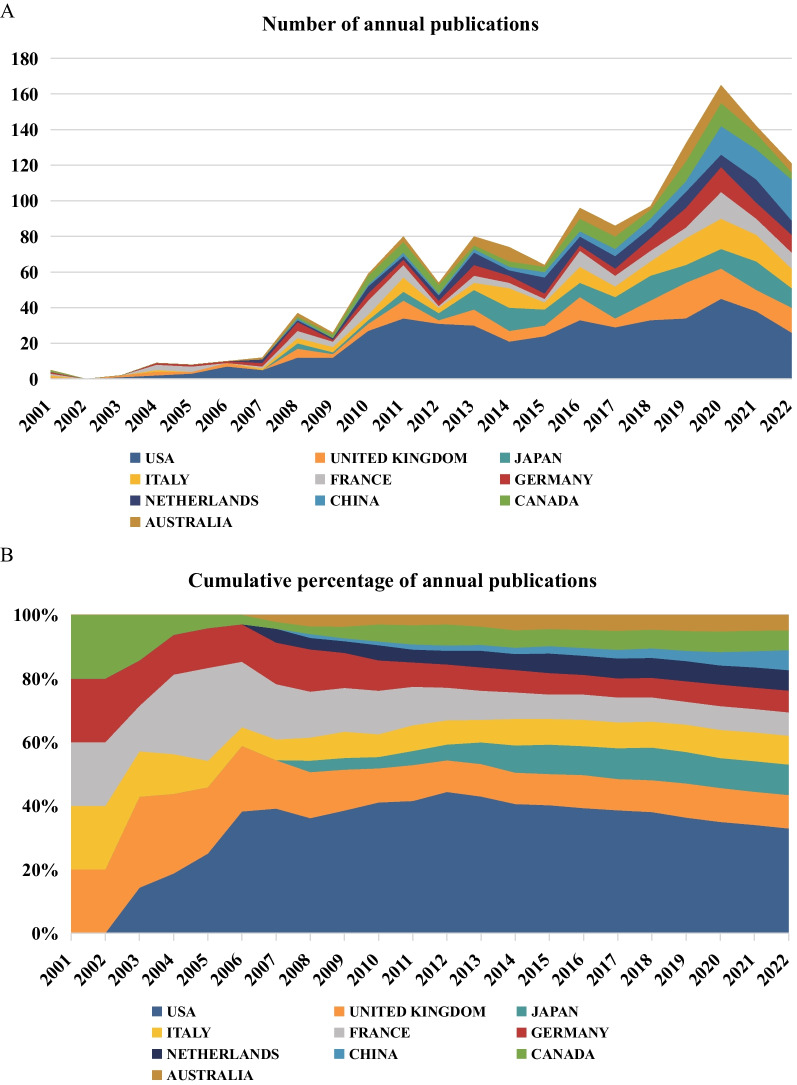
National publications in the field of residual cardiovascular risk research. **A** The number of papers published each year by the top 10 countries. **B** Proportion of accumulative published articles in the top 10 countries

A total of 66 countries and regions have participated in studies of residual cardiovascular risk. The United States is the most significant contributor of scholarly articles in this field, with the UK, Japan, Italy, and France following in that order, as evidenced by the data in Table 1. These countries have a high level of research enthusiasm and contribution to the residual risk of CVD. Moreover, the USA has demonstrated the most significant academic impact, evidenced by achieving the highest h-index relative to other countries. As depicted in Fig. 3, the USA consistently leads in terms of annual publication volume. The UK, Italy, France, Germany, and Canada were pioneers in initiating research in this field. In contrast, the Netherlands, Japan, China, and Australia commenced their research endeavors at a later stage, as compared to the aforementioned countries.

**Table 1:** Top 10 countries with the most publications

Rank	Country	NP	TC	ACI	TLS	h-index
1	USA	446	22,459	50.36	480	72
2	UK	143	5823	40.72	441	39
3	Japan	130	4070	31.31	101	30
4	Italy	123	5349	43.49	366	39
5	France	100	4452	44.52	332	33
6	Germany	92	3951	42.95	319	27
7	Netherlands	87	3122	35.89	260	28
8	China	86	846	9.84	43	16
9	Canada	84	6527	77.70	201	32
10	Australia	66	3318	50.27	158	26

*NP* number of publications, *TC* total citations, *ACI* average number of citations per item, *TLS* total link strength

**Fig. 4:**
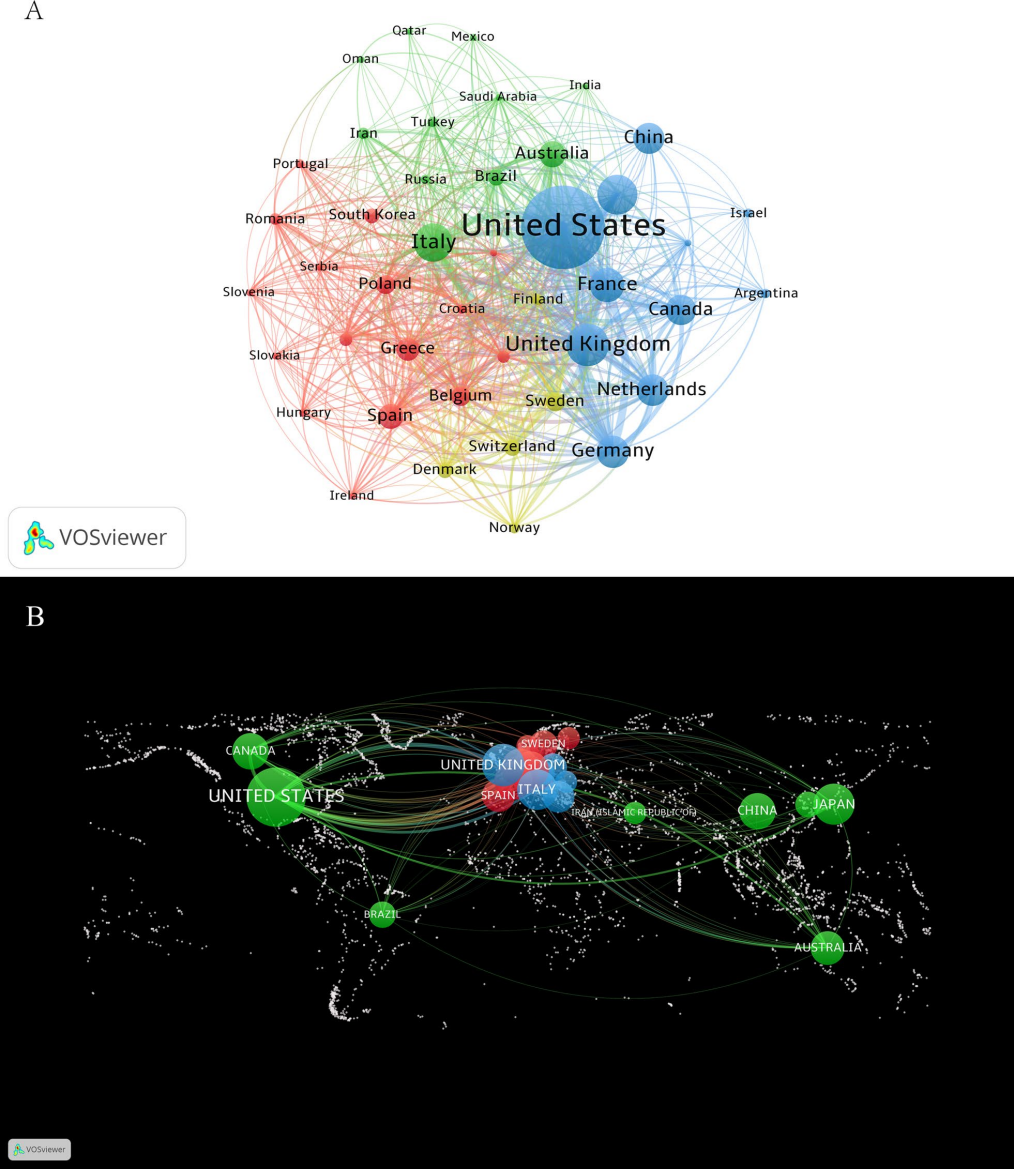
National contributions and partnerships in residual cardiovascular risk research. **A** Cooperation and clustering between countries. **B** Distribution of the main partner countries on the world map

We employed VOSviewer to analyze inter-country cooperation visually, setting the minimum number of documents per country to 5. The resulting network (Fig. 4A) displays 43 countries and regions, 4 clusters, and 612 links. The blue cluster is the largest and comprises mainly the USA, France, the UK, Germany, and Canada. The green cluster is mainly composed of Italy, Australia, Brazil, Iran, and other countries, while the red clusters mainly consist of Spain, Greece, Belgium, Poland, and South Korea. The yellow clusters are mainly Sweden, Switzerland, Denmark, Norway, and Finland. The world map of country cooperation indicates that countries with the closest cooperation are concentrated in North America, Western Europe, and East Asia (Fig. 4B), with the USA as the center of cooperation, closely collaborating with the UK, France, Japan, Italy, and others.

### Contributions of institutions

**Fig. 5:**
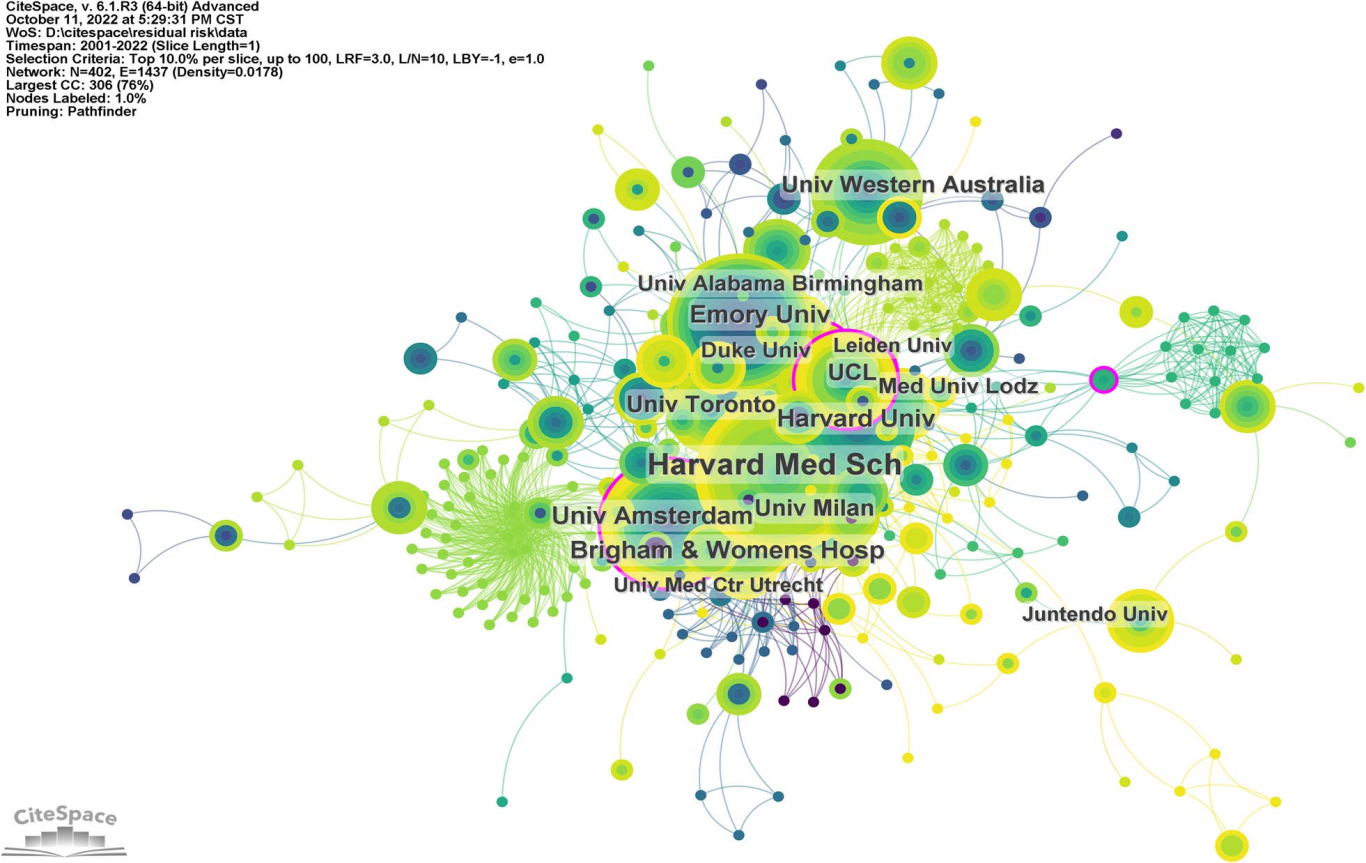
Research institution cooperation network

Research institutions are the most oriented research forces, generally with clear research directions and tasks. The views of important research institutions tend to have a strong influence. A total of 2133 institutions participated in the study of residual cardiovascular risk. CiteSpace was used to draw the institutional cooperation chart for the literature data (Fig. 5). Betweenness centrality (BC) is a measure of the importance of nodes in the network. Nodes with high centrality play the role of "communication bridges" in the network [20]. BC greater than or equal to 0.1 is highlighted with a purple outer circle in the figure. Institutions with high BC include the University of London (0.15), Leiden University (0.13), the University of Amsterdam (0.11), the National Heart Lung and Blood Institute (0.11), Kumamoto University (0.11), University of Colorado (0.1), McMaster University (0.1). In the institutional cooperation network diagram, there are 402 nodes and 1437 connections, and the network density is 0.0178. The most published institutions were Harvard Medical School (51 articles), Brigham & Women's Hospital (33 articles), University of Amsterdam (31 articles), Emory University (29 articles), and University Of Milan (12 articles) (Table 2 shows the details).

**Table 2:** The top 10 research institutions with the most published papers

Rank	Affiliations	NP	TC	ACI	Centrality	h-index
1	Harvard Medical School	51	2056	40.31	0.08	23
2	Brigham and Women's Hospital	33	2068	62.67	0.06	24
3	University of Amsterdam	31	1326	42.77	0.11	18
4	Emory University	29	2916	100.55	0.09	18
5	University of Milan	25	1626	65.04	0.08	19
6	Harvard University	24	2929	122.04	0.07	19
7	University College London	24	894	37.25	0.15	17
8	University of Western Australia	24	833	34.71	0.04	15
9	University of Toronto	22	529	24.05	0.01	13
10	University of Alabama Birmingham	21	1234	58.76	0.03	13

### Contributions of author

**Fig. 6:**
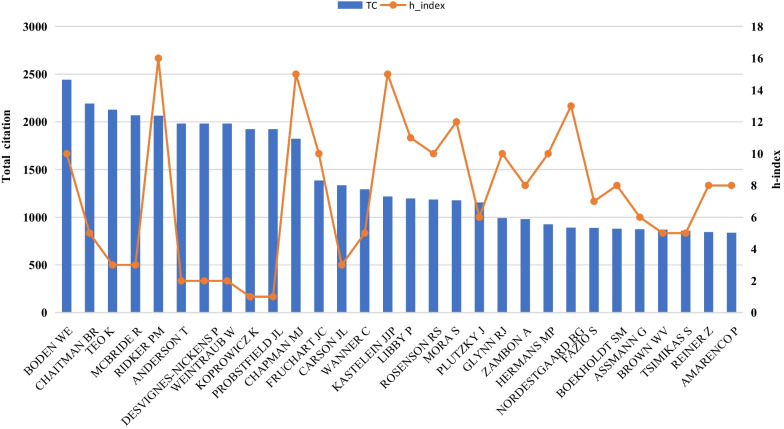
Internationally important author in the field of residual cardiovascular risk research

A total of 5067 authors contributed to the 1167 articles. Citation count is an intuitive indicator of an author's influence, although it may be influenced by their number of publications. The h-index is a composite metric used to assess scholarly accomplishment, indicating that an author has “h” number of papers each cited at least “h” times [21]. The g-index is a derivative of the h-index and is a complement to the h-index. In simple terms, the g-index means that the author has an average of “g” articles (in the h-index, which is "least", not "average") citations of no less than “g” [22]. The g-index can find authors who have published one or several highly cited articles. Generally, both the h-index and g-index are intuitive indicators of whether an author has published enough high-quality articles. We used the bibliometrix to count the author's publication volume, total citation frequency, h-index, and g-index (Table 3). According to the total citation frequency, the authors with high international influence are Boden We, Chaitman BR, Teo K, Mcbride R, and Ridker PM. Comprehensive h-index, high-influence authors also include Chapman MJ, Kastelein JJP, Nordestgaard BG, Watts GF, and Banach M. We visualize the top 30 most cited authors, as shown in Fig. 6. The bar graph represents the citation frequency, and the line graph represents the h-index. Ridker PM and Boden WE have the most citations and h-index among many scholars. They have contributed to the study of residual cardiovascular risk.

**Table 3:** Authors with the top 10 Total citations

Rank	Author	NP	TC	h-index	g-index
1	Boden WE	12	2440	10	12
2	Chaitman BR	5	2190	5	5
3	Teo K	3	2125	3	3
4	Mcbride R	4	2067	3	4
5	Ridker PM	18	2062	16	18
6	Anderson T	2	1982	2	2
7	Desvignes-Nickens P	2	1982	2	2
8	Weintraub W	2	1982	2	2
9	Koprowicz K	1	1923	1	1
10	Probstfield JL	1	1923	1	1

### Contributions of journals

Journals serve as the primary medium for the sharing and dissemination of academic work. We used VOSviewer to identify journals with a high volume of publications and significant impact in the field. To evaluate a journal's impact, key indicators such as the number of citations, impact factor (IF), and Journal Citation Reports (JCR) quartile were analyzed. A total of 386 journals were found to have published articles on residual cardiovascular risk. Table 4 and Additional file 1: Table 1 present the top 10 journals based on the number of publications and citations. Out of the top 10 most cited journals, 6 have an IF of more than 10, and 9 are in the Q1 JCR. Notably, the New England Journal of Medicine (NEJM), with only a small number of published research papers, has garnered the most citations (176.079, Q1), followed by the Journal of the American College of Cardiology (JACC) with an IF of 27.203 (Q1) and 1757 citations, Atherosclerosis with an IF of 6.847 (Q1) and 1554 citations, the European Heart Journal (EHJ) with an IF of 35.855 (Q1) and 1535 citations, and Circulation with an IF of 39.918 (Q1) and 1275 citations. It is noteworthy that five of the top 10 most cited journals are also among those with the most publications, including Atherosclerosis, American Journal of Cardiology, Journal of Clinical Lipidology, JACC, and EHJ. These journals have a strong influence and play an important role in academic communication. Among the top 10 most published journals, Atherosclerosis had the highest number of publications, followed by Current Atherosclerosis Reports, American Journal of Cardiology, Current Opinion in Lipidology, Journal of Clinical Lipidology, JACC, EHJ, Journal of the American Heart Association (JAHA), Cardiovascular Diabetology, and International Journal of Cardiology.

**Table 4:** Top 10 journals with the most publications in the field of residual cardiovascular risk

Rank	Journals	JCR	IF	NP	TC	h-index
1	Atherosclerosis	Q1	6.847	40	1554	20
2	Current Atherosclerosis Reports	Q1	5.967	34	814	12
3	American Journal of Cardiology	Q3	3.133	32	1098	17
4	Current Opinion in Lipidology	Q2	4.616	21	431	12
5	Journal of Clinical Lipidology	Q1	5.365	21	833	10
6	Journal of the American College of Cardiology	Q1	27.203	20	1757	18
7	European Heart Journal	Q1	35.855	19	1535	16
8	Journal of the American Heart Association	Q1	6.106	19	452	10
9	Cardiovascular Diabetology	Q1	8.949	18	811	12
10	International Journal of Cardiology	Q2	4.039	16	398	11

**Fig. 7:**
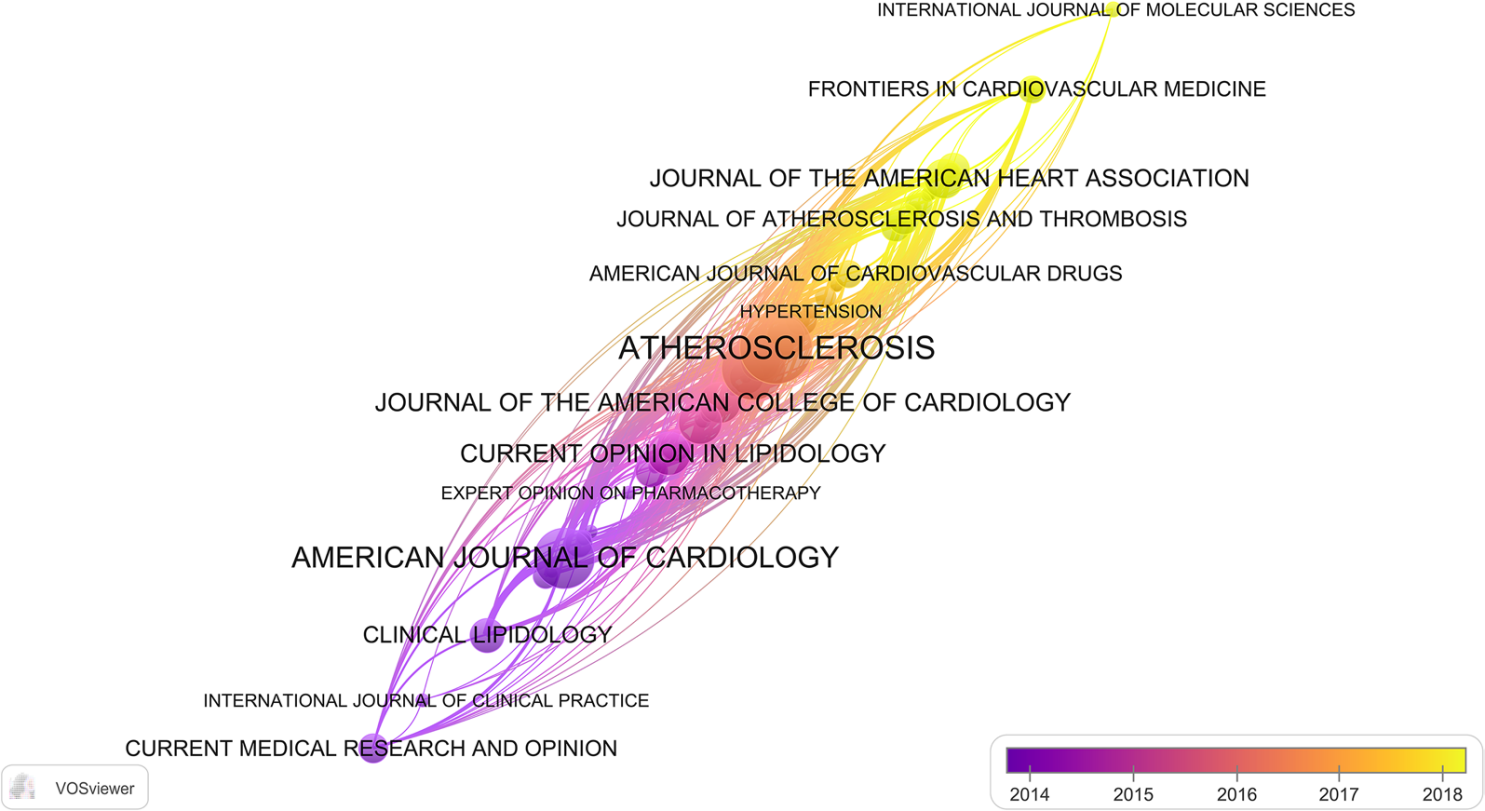
The number and time trend of journal publications in the field of residual cardiovascular risk

VOSviewer visualizes the volume of journal publications and their average publication year (Fig. 7). The size of each node in the graph corresponds to the number of published articles on a specific topic. The node colors represent the average publication year, with warmer colors denoting more recent publications and cooler colors indicating earlier ones. Atherosclerosis has a high number of publications, and the average publication year is in the middle of these journals, indicating that the journal continues to pay attention to the research on residual cardiovascular risk. Current Medical Research and Opinion, International Journal of Clinical Practice and Clinical Lipidology reported early findings on residual cardiovascular risk. International Journal of Molecular Sciences, Frontiers in Cardiovascular Medicine, and JAHA have been more interested in this field in recent years.

**Fig. 8:**
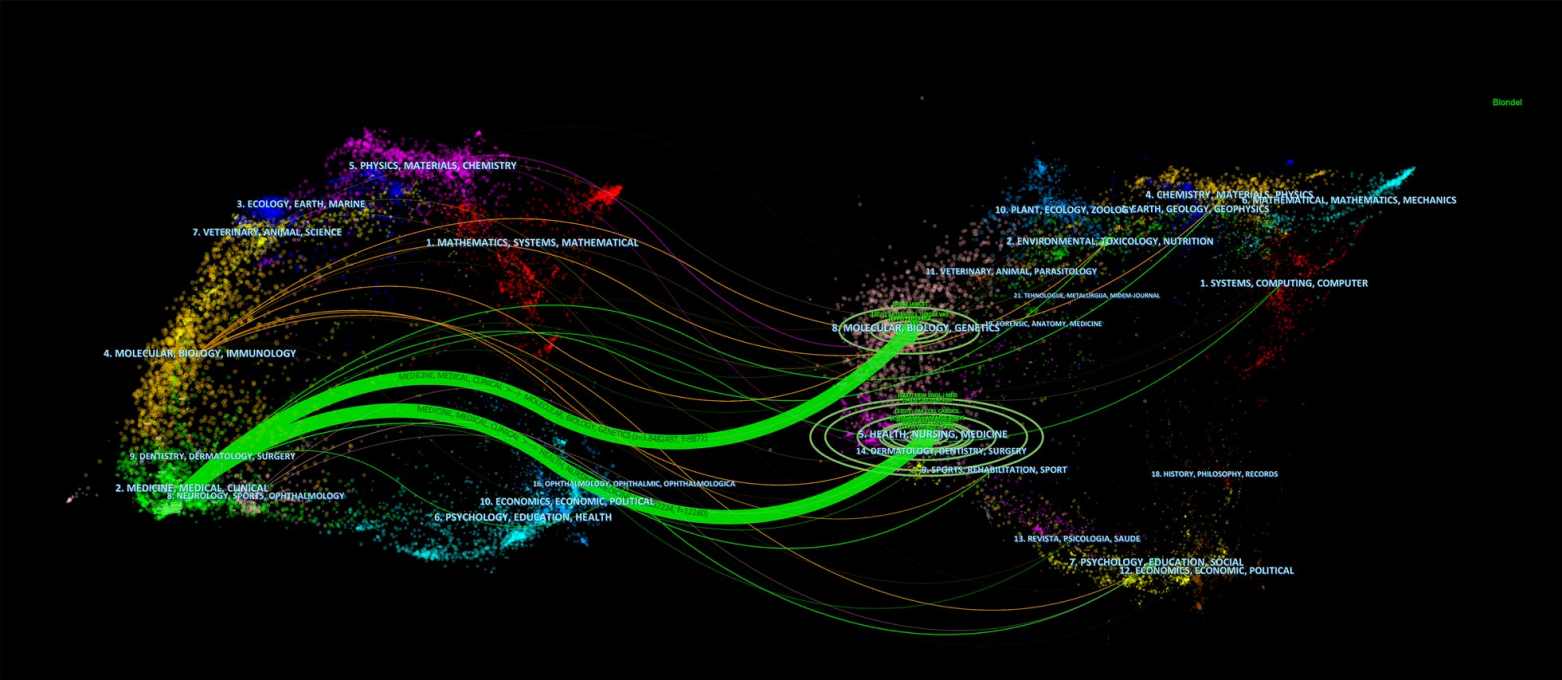
The dual-map overlay of journals related to residual cardiovascular risk research

We utilized a dual-map overlay to display the distribution of topics and flow of knowledge in studies on residual cardiovascular risk. The left side of the graph represents the citing journals and the right side represents the cited journals. The wavy lines depict the citation links. The color represents the disciplinary clustering of the journal [23]. From Fig. 8, we can see the two most dominant paths (green path). The source journals of the citing literature are mainly distributed in medicine, medical and clinical disciplines. The citing literature in these directions is divided into two main data streams. The first data stream shows that the cited literature is mainly from the disciplinary work of health, nursing, and medicine. Major journals include NEJM, Circulation, and the JACC. The second data stream represents the source journals of the cited articles in the disciplines focused on molecular, biology, and genetics, with representative journals including The Lancet, Arteriosclerosis, Thrombosis, and Vascular Biology, and Journal of Lipid Research.

### Keyword analysis

**Fig. 9:**
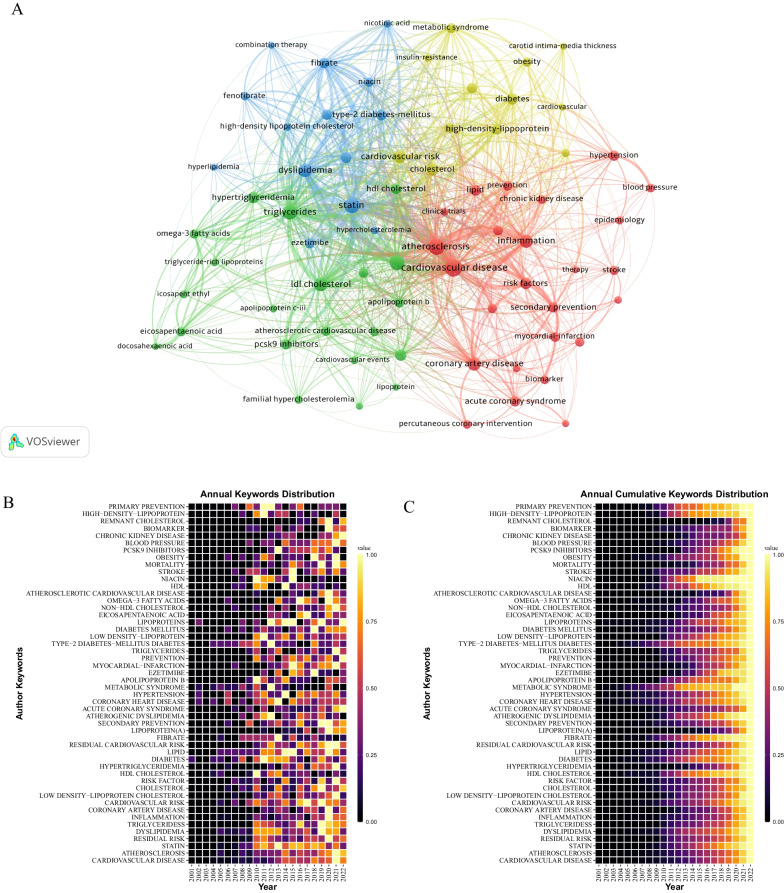
High-frequency keywords. **A** Keywords co-occurrence network. **B** Annual change of high-frequency keywords. **C** Annual cumulative distribution of high-frequency keywords

Keywords serve as a condensed summary of the fundamental content of a research article and wield significant influence in the widespread communication of scholarly outcomes. Analyzing the keywords of a paper can reveal the themes of the article. The keyword co-occurrence network diagram shows the associations among keywords (Fig. 9A). The size of each node reflects the frequency of the corresponding keyword in the literature. The thicker the connection between the two keywords, the more frequently the two keywords co-occurrence. Keyword clusters are divided into four categories: red, yellow, blue, and green. The red cluster includes CVD, atherosclerosis, inflammation, chronic kidney disease, coronary artery disease (CAD), acute coronary syndrome (ACS), blood pressure, and stroke. The yellow cluster includes cardiovascular risk, cholesterol, high-density lipoprotein (HDL), diabetes, metabolic syndrome, obesity, and insulin resistance. Keywords in the blue cluster include statin, dyslipidemia, ezetimibe, fibrates, fenofibrate, niacin, and combination therapy. The green cluster includes triglycerides (TG), hypertriglyceridemia, LDL-C, apolipoprotein b (ApoB), lipoprotein, omega-3 fatty acids, icosapent ethyl, PCSK9 inhibitors, eicosapentaenoic acid (EPA), docosahexaenoic acid (DHA).

The first appearance time of keywords can roughly reflect the order of the research topic. High-frequency keywords in a certain period reflect the research hotspots at that time. Figure 9B shows the annual change of the top 50 keywords. The warmer the color, the more frequently that keyword appears in the year. Diabetes is the first keyword to appear in 2001. In addition, the early keywords also include CAD, hypertension, lipoproteins, and metabolic syndrome. However, researchers have paid less attention to metabolic syndrome in recent years. The same goes for niacin, which was mostly studied from 2010 to 2012. In contrast, studies of TG, inflammation, lipoprotein (a)[LP(a)], secondary prevention, blood pressure, and biomarker have become more popular in recent years. Notably, academics have been enthusiastic about statins since 2010.

Figure 9C shows the heat map plotted by the accumulated frequency of keywords. The earlier the color turns yellow means the earlier the keyword matures. Late-maturing keywords represent the frontier of the research, such as hypertriglyceridemia, LP(a), ACS, myocardial-infarction, EPA, atherosclerotic cardiovascular disease (ASCVD), mortality, chronic kidney disease, and remnant cholesterol.

### Highly cited articles

The quantity of citations received by a research article serves as the gold standard for evaluating its academic worth. Highly cited papers tend to attract more attention from scholars. Additional file 2: Table 2 and Table 5 summarize the top 10 articles in terms of global citations (GC) and local citations (LC). GC refers to the total number of citations for this article in the Web of Science database. LC are the number of citations by the 1167 articles included in this study [24]. Articles with high citations are important documents in the field. Because the included literature search strategy is consistent, local literature can be considered your research peers. Therefore, LC represent peer recognition and are more representative of high-quality literature in this field than GC. The following is the main content of the top 10 articles in LC.

**Table 5:** Top 10 articles by LC

Rank	Title	Journal	Author	Year	LC	GC
1	Niacin in patients with low HDL cholesterol levels receiving intensive statin therapy	New Engl J Med	Boden WE	2011	153	1923
2	The Residual Risk Reduction Initiative: a call to action to reduce residual vascular risk in dyslipidaemic patient	Diabetes Vasc Dis Res	Fruchart JC	2008	42	212
3	Residual cardiovascular risk despite optimal LDL cholesterol reduction with statins: the evidence, etiology, and therapeutic challenges	Curr Atheroscler Rep	Sampson UK	2012	41	198
4	HDL cholesterol and residual risk of first cardiovascular events after treatment with potent statin therapy: an analysis from the JUPITER trial	Lancet	Ridker PM	2010	39	178
5	A Test in Context: Lipoprotein(a): Diagnosis, Prognosis, Controversies, and Emerging Therapies	J Am Coll Cardiol	TSIMIKAS S	2017	34	405
6	Determinants of residual risk in secondary prevention patients treated with high- versus low-dose statin therapy: the Treating to New Targets (TNT) study	Circulation	Mora S	2012	33	108
7	Lipoprotein(a) concentrations, rosuvastatin therapy, and residual vascular risk: an analysis from the JUPITER Trial (Justification for the Use of Statins in Prevention: an Intervention Trial Evaluating Rosuvastatin)	Circulation	Khera AV	2014	31	229
8	Residual macrovascular risk in 2013: what have we learned?	Cardiovasc Diabetol	Fruchart JC	2014	31	124
9	Rationale and design of the Pemafibrate to Reduce Cardiovascular Outcomes by Reducing Triglycerides in Patients with Diabetes (PROMINENT) study	Am Heart J	Pradhan AD	2018	31	179
10	Meta-analysis of the effect of nicotinic acid alone or in combination on cardiovascular events and atherosclerosis	Atherosclerosis	Bruckert E	2010	26	216

The article by Boden et al. [25] has the most citations. This article demonstrated that in patients with ASCVD and LDL-C less than 70 mg per deciliter, the addition of niacin to statin therapy did not reduce residual cardiovascular risk, but HDL and TG levels were significantly improved [25].

The article "The Residual Risk Reduction Initiative: a call to action to reduce residual vascular risk in dyslipidaemic patient" was the second most cited paper [26]. The Residual Risk Reduction Initiative (R3i) is an organization of many prominent basic and clinical experts who focus on complications in patients with atherosclerosis. The article defines atherogenic dyslipidemia as an imbalance between ApoB and apolipoprotein AI. Five years later, R3i published another article (ranked 8) that further highlighted the use of non-HDL cholesterol as a key target for treatment decisions related to lipid-related residual cardiovascular risk [27]. They also updated the evidence on fibrate, niacin, and omega-3 fatty acids combined with statin therapy, as well as some emerging treatments.

The third most cited article was a review on residual cardiovascular risk [3]. This article proposes that reducing absolute risk by improving lifestyle is the core strategy for addressing residual cardiovascular risk caused by HDL cholesterol (HDL-C)/TG.

Articles ranked 4 and 7 are clinical studies based on the “JUPITER” trial and published in The Lancet and Circulation, respectively [28, 29]. The two studies demonstrated that HDL-C is not a contributing factor to the increased residual risk of CVD in patients receiving effective statin therapy, while LP(a) is.

The fifth-ranked article, published in JACC in 2017, summarizes the research progress and controversies in Lp(a) [30]. The sixth-ranked article was a randomized controlled study targeting determinants of residual cardiovascular risk in addition to lipids [31]. The findings showed that among patients receiving statin therapy for secondary prevention of coronary heart disease, multiple factors affected residual risks, such as baseline lipoprotein, increased body mass index, smoking, diabetes, and hypertension. Therefore, multiple interventions should be advocated to reduce risk. The article ranked 9 analyzed the design rationale for the Pemafibrate Reduced Cardiovascular Outcomes by Reducing Triglycerides in Diabetic Patients (PROMINENT) study [32]. The PROMINENT trial is a landmark international multicenter cardiovascular outcomes trial that promises to provide new treatment options for people with diabetes with high TG and low HDL-C. The final article is a meta-analysis which identified a beneficial impact of niacin, either alone or in combination, on cardiovascular events and the progression of atherosclerosis. [33]

### Co-citation references

Co-citation is a metric that assesses the correlation between two documents by analyzing the frequency at which they are referenced collectively by other publications. It is a way to identify the similarity between two documents based on their references rather than their content [34–36]. Through the examination of the frequency at which two articles are co-cited, scholars can pinpoint clusters of highly pertinent articles, which can form the basis of a specific research area. In addition, co-citation analysis allows studies to determine the development process and trends in the field. BC greater than 0.1 were the articles published by Cohen JC 2006 and Bonaca MP 2018, which formed a co-citation relationship with several articles in the field.

Table 6 lists the 10 articles with the highest number of co-citations. Baigent C published two meta-analyses on the efficacy and safety of intensive lipid-lowering in 2005 and 2010 [37, 38]. Keech A and Ginsburg HN published a randomized controlled trial of fenofibrate on cardiovascular risk in patients with type 2 diabetes in 2005 and 2010, respectively [39, 40]. Barter PJ published the article "Effects of torcetrapib in patients at high risk for coronary events" in 2007 [41]. However, the experiment was terminated due to excessive adverse effects. Cannon CP mainly studies the effect of intensive lipid-lowering after ACS [42, 43]. Sabatine MS published a randomized, double-blind, placebo-controlled trial of evolocumab, a fully human PCSK9 monoclonal antibody, in 2017 [44]. The article by Ridker PM has also received significant attention for demonstrating the potential of statins in reducing the incidence of major cardiovascular events in patients with elevated C-reactive protein but no hyperlipidemia [45]. Prof. Boden WE's study on niacin is not only highly co-cited, but it is also the most cited article in 1167 articles [25]. This indicates that this is a landmark study on cardiovascular residual risk.

**Table 6:** Top 10 articles with the highest number of co-citations

Rank	Title	Journal	Author	Year	LC
1	Effects of combination lipid therapy in type 2 diabetes mellitus	New Engl J Med	Ginsberg HN	2010	177
2	Efficacy and safety of more intensive lowering of LDL cholesterol: a meta-analysis of data from 170,000 participants in 26 randomised trials	Lancet	Baigent C	2010	174
3	Efficacy and safety of cholesterol-lowering treatment: prospective meta-analysis of data from 90,056 participants in 14 randomised trials of statins	Lancet	Baigent C	2005	170
4	Effects of torcetrapib in patients at high risk for coronary events	New Engl J Med	Barter PJ	2007	157
5	Niacin in patients with low HDL cholesterol levels receiving intensive statin therapy	New Engl J Med	Boden WE	2011	153
6	Effects of long-term fenofibrate therapy on cardiovascular events in 9795 people with type 2 diabetes mellitus (the FIELD study): randomised controlled trial	Lancet	Keech A	2005	138
7	Evolocumab and Clinical Outcomes in Patients with Cardiovascular Disease	New Engl J Med	Sabatine MS	2017	135
8	Ezetimibe Added to Statin Therapy after Acute Coronary Syndromes	New Engl J Med	Cannon CP	2015	131
9	Intensive versus moderate lipid lowering with statins after acute coronary syndromes	New Engl J Med	Cannon CP	2004	124
10	Rosuvastatin to prevent vascular events in men and women with elevated C-reactive protein	New Engl J Med	Ridker PM	2008	120

**Fig. 10:**
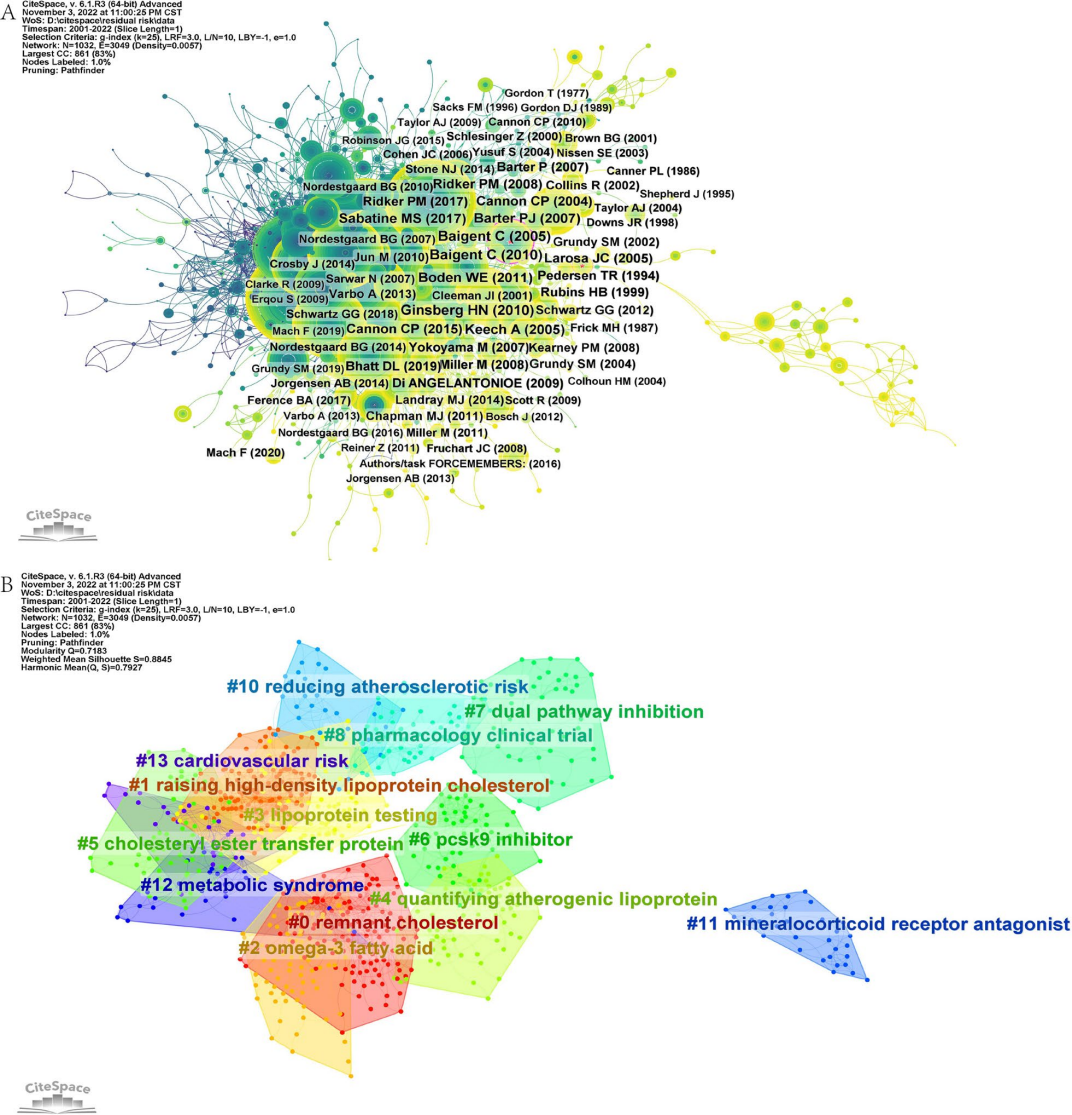
Reference co-citation analysis map. **A** Reference co-citation network. **B** Reference co-citation clustering

CiteSpace was employed to establish a co-citation network of references, as depicted in Fig. 10A, which comprised 1032 nodes and 3049 links. Colors in the network ranged from cold to warm, representing early to late time periods. The size of the node was proportional to the number of citations. Initially, the left side of the network was dominated by a small number of highly cited studies. However, in recent years, research directions have become more diversified, resulting in a dispersed network structure. The clustering analysis of the co-citation literature is presented in Fig. 10B, revealing a total of 13 clusters. Combining this with Fig. 10A, it can be observed that in the early stages of residual risk research, the co-citation network was relatively dense. The main research topics during this period were focused on reducing atherosclerotic risk, pharmacology clinical trials, cardiovascular risk, raising HDL-C, lipoprotein testing, cholesteryl ester transfer protein, and metabolic syndrome, among others. In recent years, the research has expanded to include topics such as pathway inhibition, PCSK9 inhibitors, quantifying atherogenic lipoproteins, remnant cholesterol, omega-3 fatty acids, and mineralocorticoid receptor antagonists.

### Burst detection

**Fig. 11:**
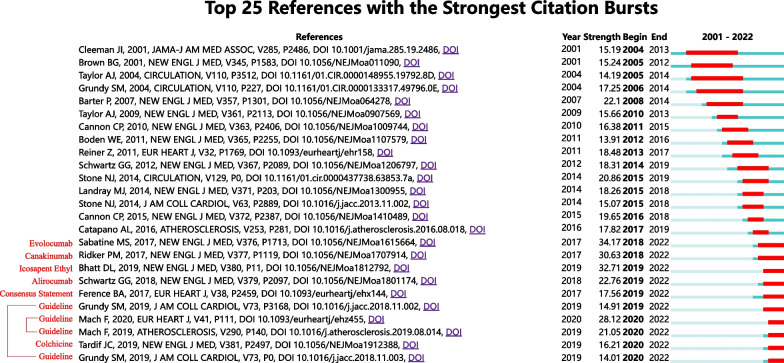
Top 25 references with the strongest citation bursts

Figure 11 represents the results of burst detection based on the references co-citation network. Cleeman JI, Brown BG, Taylor AJ, Grundy SM's article received scholarly attention in the early. Their articles have a relatively long burst duration [46–49]. Their research has, to some extent, laid the groundwork for the development of cardiovascular residual risk.

The articles with burst periods lasting until 2022 represent current research hotspots. A total of 10 articles were eligible, 5 of which were guidelines or expert consensus (red lines connect to the same guidelines). In 2017, the European Atherosclerosis Society published a consensus statement. They consistently demonstrated that LDL causes ASCVD by evaluating many different types of clinical and genetic studies [50]. In 2018, the American Heart Association (AHA) /American College of Cardiology (ACC) released new cholesterol management guidelines. The key highlights of the guidelines are: 1) specifying a target of ≥ 50% reduction in LDL-C for patients with clinical ASCVD and 2) recommending an LDL-C reduction to less than 1.8 mmol/L for very high-risk ASCVD patients, using statin therapy in combination with ezetimibe or PCSK9 inhibitors [51].

Less than a year later, the European Society of Cardiology (ESC) / the European Atherosclerosis Society (EAS) also published guidelines for the management of dyslipidemia. Both guidelines emphasize lowering LDL-C ratios to reduce cardiovascular risk, including the use of statins and non-statin drugs. Furthermore, they both emphasized the importance of lifestyle interventions. The main differences between the two guidelines are in the methods of risk assessment, the definition of high-risk groups, and treatment goals. Compared with the 2016 ESC/ECA guidelines for the management of dyslipidemia, the 2019 guidelines recommend the use of n-3 polyunsaturated fatty acids combined with statins to lower TG in high- and very-high-risk patients. Notably, there are no recommendations for ASCVD anti-inflammatory therapy [52, 53].

The other 5 articles are clinical studies, all published in NEJM. Professor Sabatine MS's article has been discussed earlier. [44] Another study on PCSK9 inhibitors was published in 2018. This study found that in patients with previous ACS receiving high-intensity statin therapy, treatment with alirocumab resulted in a lower incidence of recurrent ischemic cardiovascular events compared to those treated with a placebo [54].

Ridker PM and Tardif JC reported the results of Canakinumab Antiinflammatory Thrombosis Outcomes Study (CANTOS) and Colchicine Cardiovascular Outcomes Trial (COLCOT) in 2017 and 2019, respectively. The first CANTOS trial to use anti-inflammatory drugs in patients with CHD, using the interleukin 1ß monoclonal antibody canakinumab, showed that compared with the placebo group, the risk of cardiovascular events was reduced by 15%. However, it also slightly increased the incidence of fatal infections [55]. In the COLCOT trial, patients with recent myocardial infarction who were treated with colchicine in addition to appropriate drug therapy had a significantly lower incidence of cardiovascular events compared to those who received a placebo [56].

Professor Bhatt DL reported the research results of Reduction of Cardiovascular Events with Icosapent Ethyl-Intervention Trial (REDUCE-IT) in 2019. Patients who were treated with 4 g daily of icosapent ethyl had a significantly lower incidence of major adverse cardiovascular events compared to those who received a placebo, with a risk reduction of 25% [57].

## Discussion

### General information

In this study, we analyzed the generation and development of residual cardiovascular risk using a bibliometric approach. Research on residual cardiovascular risk began in the early twenty-first century, and it showed a steady upward trend in general. In the first 10 years, the number of studies has increased slowly, indicating that research on residual cardiovascular risk was still in its initial stages and lacking a solid research foundation. From 2010 to 2017, the annual number of articles remained stable, suggesting that residual cardiovascular risk was in a continuous exploratory phase. The number of articles has increased rapidly since 2018, with an increasing number of researchers focusing on the impact of residual risk on cardiovascular patients. This indicates that this area of research is maturing. In the coming years, residual cardiovascular risk is likely to become a hot topic and research direction in the field of cardiovascular studies.

In the realm of residual cardiovascular risk research, the USA has consistently held a leading position, with the highest number of published papers and citation frequency. The average citations per item (ACI) for US papers is 50.36, indicating the high impact and quality of its scientific output. Furthermore, the USA has an extensive academic collaboration network, with a total link strength (TLS) of 480, significantly surpassing other countries, which underscores its central role in international scientific collaboration. The UK, Japan, Italy, and France follow closely, each with over 100 published papers and several thousand citations, demonstrating their substantial research contributions and influence in the field of residual cardiovascular risk. Their relatively high ACI and h-index further validate the quality of their research and international standing.

In the realm of residual cardiovascular risk research, the USA has consistently held a leading position, with the highest number of published papers and citation frequency. The ACI for US papers is 50.36, indicating the high impact and quality of its scientific output (Table 1). Furthermore, the USA has an extensive academic collaboration network, with a TLS of 480, significantly surpassing other countries, which underscores its central role in international scientific collaboration. The UK, Japan, Italy, and France follow closely, each with over 100 published papers and several thousand citations, demonstrating their substantial research contributions and influence in the field of residual cardiovascular risk. Their relatively high ACI and h-index further validate the quality of their research and international standing. In comparison, China, despite having a similar number of published papers (86), shows a considerable gap in total citations (846) and ACI (9.84). This discrepancy may reflect China's untapped potential in international collaboration and the need to enhance the international impact of its research findings. Therefore, looking to the future, there is a pressing need to intensify academic cooperation on a global scale, to pool the collective wisdom of various nations and jointly drive the advancement and innovation in research on residual cardiovascular risk.

The US leadership in residual cardiovascular risk was similarly demonstrated in the institutional analysis. Of the top 10 institutions, 50% were based in the USA, while the other half were located in five different countries. Further analysis revealed that universities were the most prominent institutions and the backbone of the research. Harvard Medical School, Brigham & Women's Hospital, and the University of Amsterdam have a strong passion for residual cardiovascular risk research. The University of London, Leiden University, University of Amsterdam, National Heart Lung and Blood Institute, Kumamoto University, University of Colorado, and McMaster University played an important role in the exchange and dissemination of scholarship.

More than 5,000 authors contribute to residual cardiovascular risk. Ridker PM and Boden WE are among the most influential scholars. Ridker PM is a cardiologist and epidemiologist at Harvard Medical School, known for his pioneering work on inflammation and CVD [55, 58], including the discovery of the role of C-reactive protein as an independent risk factor [45, 59]. He has also demonstrated the effectiveness of low-dose aspirin in preventing heart attacks and strokes [60]. In addition, his research on genetics and personalized medicine has revealed how genetic variants affect CVD risk, providing new diagnostic and treatment strategies [61–64]. His contributions have greatly improved our understanding of the pathophysiology, prevention and treatment of CVD. Dr. William E. Boden is an American CVD specialist and clinical trialist currently working at Boston University School of Medicine. Dr. Boden has led several large-scale clinical trials, including the classic COURAGE trial (Clinical Outcomes Utilizing Revascularization and Aggressive Drug Evaluation). His research has provided important clinical guidelines for the treatment and prevention of CVD [65].

Many high-quality and high-impact journals have a strong focus on residual cardiovascular risk. A total of 386 journal articles were included in the study, with the most prolific being Atherosclerosis, American Journal of Cardiology, and Current Opinion in Lipidology. The 10 most published journals published 20.5% of the articles. NEJM, JACC, Atherosclerosis, and EHJ, were the most concerning journals, and their citations are more than 1500. These 1167 articles have been cited a total of 36,279 times. The top 10 most cited journals accounted for 36.1% of the total citations. This suggests that these journals include most of the core literature on residual cardiovascular risk research. Atherosclerosis is the most worthy journal for scholars, and it continues to pay high attention to research in this field.

The citation and cited relationship of journals find a convergence of disciplinary knowledge. That is, knowledge from the molecular, biological, and genetic fields, together with knowledge from health and nursing, converge into the medical and clinical fields.

### Hotspots and frontiers

Our study employed keyword analysis, co-citation analysis, and burst detection to identify active and emerging areas in residual cardiovascular risk research. As previously mentioned, keywords in scientific literature are carefully chosen by authors to highlight the core concepts and themes of their research. Within a given field, frequently occurring keywords often point to the hotspots of research [66]. Moreover, by analyzing the co-citation relationships between documents, we can identify which publications play a central role in academic discourse, namely those that are often cited together to support subsequent research [67]. These core publications typically represent significant research findings or foundational theories within the field. Hence, co-citation analysis helps to reveal the knowledge structure and research hotspots within a research domain. In bibliometrics, burst detection is commonly used to identify a significant increase in the citation frequency of a particular keyword or publication within a specific timeframe [68, 69]. Such an increase indicates that the research community's interest in a topic is rapidly growing, and the topic may represent an emerging research frontier or an upcoming trend. Burst detection allows researchers to promptly capture new dynamics and shifts in scientific research. In summary, by employing these three methods to analyze the literature data, we aim to uncover the hotspots and frontier trends concerning residual cardiovascular risk research.

### Hotspots

The clustering of keywords led to 4 main research directions for residual cardiovascular risk. The first is for residual risk-related complications (red cluster), including CVD, cardiovascular events, atherosclerosis, ACS, myocardial infarction, and stroke. The second research area is related to cardiovascular risk factors (yellow cluster), with a focus on diabetes, cholesterol, metabolic syndrome, obesity, and insulin resistance. The third is pharmacological prevention of residual cardiovascular risk (blue cluster), with statin being the predominant therapeutic agent and others including ezetimibe, fibrates, fenofibrate, niacin, and combination therapy. The fourth research direction is a hot research area in recent years (green cluster) and includes hypertriglyceridemia, ApoB, LP(a), omega-3 fatty acids, icosapent ethyl, PCSK9 inhibitors, EPA, DHA.

Local high-cited literature and highly co-citation references also reflect the hot spots of research. The top 10 LC were mainly related to, a review of residual cardiovascular risk, risk factors, clinical studies and meta-analyses of niacin, and clinical studies of HDL-C and LP(a). Intensive lipid-lowering and combined lipid-lowering were the most important topics in the top 10 co-citation references. In addition, there was one study related to C-reactive protein. However, it often takes years for an article to go from publication to being cited multiple times. Therefore, these research topics are lagging and cannot represent the frontier of research. This is also reflected in their average publication time (TOP 10 LC per Year = 2012.6, TOP 10 co-citation references per Year = 2009.2).

### Frontiers

The frontiers are summarized from the trend of keywords popularity, references co-citation clustering, and burst analysis. The current frontiers of residual cardiovascular risk are as follows:

### Comprehensive lipid-lowering

Previous studies have demonstrated that lowering LDL-C reduces cardiovascular events [70–72]. The management of blood lipid levels primarily focuses on controlling LDL-C, which has seen relatively successful outcomes. With updated guidelines and new lipid-lowering drugs, it is an era of very low LDL-C [73, 74]. However, multiple studies suggest that focusing only on LDL-C has limitations [75]. Residual cardiovascular risk is partly explained by residual lipid factors.

Cluster analysis of co-citation references has identified remnant cholesterol as a key research area, as evidenced by its placement in cluster 0#. Remnant cholesterol refers to the cholesterol content found in TG-rich lipoproteins, such as very low-density lipoproteins and intermediate-density lipoproteins [76]. Remnant cholesterol is associated with multiple cardiovascular risks from genetics, observational studies, and clinical intervention studies [77–81]. Residual cholesterol may serve as an independent predictor of multiple CVD, and its detection may help identify potential CVD not reflected by LDL-C [82, 83].

Therapeutic drugs for remnant cholesterol are also current research hotspots. Fibrate drugs, which act by activating PPARα, are lipid-lowering agents that reduce triglyceride levels and increase HDL cholesterol levels [84]. Given that a residual risk, primarily driven by high triglycerides and low HDL-C levels, persists, supplementing statin therapy with fibrates could potentially benefit patients carrying this residual risk [85]. While fibrates do exhibit certain preventive and therapeutic effects against atherosclerosis and are advantageous in treating hypertriglyceridemia [86], their usage is less widespread than statins due to side effects such as muscle and liver toxicity, gastrointestinal discomfort, and other potential adverse reactions [87]. Moreover, previous research has suggested that fenofibrate, a commonly used fibrate, does not significantly reduce cardiovascular events, further complicating the decision-making process regarding their clinical application.[39, 88] The 2020 American Diabetes Association (ADA) standard of care for diabetes no longer recommends fibrates to reduce cardiovascular risk [89]. More importantly, the recently held AHA 2022 announced the results of the PROMINENT trial: pemafibrate reduced TG levels in patients with type 2 diabetes, but did not reduce the risk of cardiovascular events and death [90]. In contrast, there have been some breakthroughs in the area of Omega-3 fatty acids. The REDUCE-IT study demonstrated a significant reduction in the incidence of cardiovascular events in patients with elevated TG levels who were already receiving statin therapy, following treatment with Icosapent Ethyl [57]. Subgroup analysis showed that the same results were obtained in the secondary prevention population [91]. These results suggest that Icosapent Ethyl may be an effective strategy to further reduce cardiovascular risk in high-risk patients beyond standard statin therapy.

Cluster #6 is PCSK9 inhibitor. PCSK9 is a liver-secreted glycoprotein that increases levels of LDL-C in the blood by degrading low-density lipoprotein receptors. PCSK9 inhibitors have been effective in lowering LDL-C levels and have shown potential in reducing the risk of CVD in a series of studies. Burst analysis indicates that research on Evolocumab and Alirocumab continues to attract academic interest. These are the two PCSK9 inhibitors currently approved for use. Evolocumab can also improve other lipid parameters, including Lp(a) levels, which is something statins cannot achieve in reducing residual cardiovascular risk [92]. Moreover, alirocumab not only affects lipid levels but may also offer additional cardiovascular benefits by reducing the activity of inflammatory cytokines (such as IL-18, IL-6, TNF-α) and other molecules associated with vascular inflammation (such as MMP-2, OPN, OPG) [93–95]. The advent of PCSK9 inhibitors provides clinicians with more options. They are often used in combination with statins for patients who have adverse reactions to statins or in whom the maximum tolerated dose of statins does not adequately control lipid levels. PCSK9 inhibitors are generally safe, with the primary safety concern being the potential reduction in vitamin E levels [96]. In addition, some new drugs have shown great potential [97]. Inclisiran is a small interfering RNA (siRNA) drug that inhibits PCSK9. It has been approved by the FDA for use in adult patients with ASCVD and heterozygous familial hypercholesterolemia [9, 98]. Inclisiran has a long-term LDL-lowering effect (6 months), so patients may have higher adherence. It is worth noting that the high price of PCSK9 inhibitors is a potential problem [99].

Keyword analysis revealed that LP(a) is also an important topic for current research. Epidemiological, Mendelian, and genomic studies confirm that elevated Lp(a) levels are an independent risk factor for a variety of CVD [100, 101]. Lp(a) is the only single genetic risk factor for AS reported to date and is one of the potential targets for reducing residual cardiovascular risk [102]. There is evidence that a substantial reduction of LP(a) can significantly prevent cardiovascular events [103], but current therapies targeted to reduce LP(a) are limited. Unlike traditional risk factors, there is a lack of strong evidence that diet or exercise can reduce LP(a) [104, 105]. Statins and ezetimibe have been shown not to reduce LP(a) [106], and lipoprotein apheresis is not an appropriate approach [107]. Although PCSK9 inhibitors and lipoprotein replacement can partially reduce LP(a) [108], there is still a lack of cost-effective drugs. Drugs that directly target LP(a) are the most promising therapies, mainly including antisense oligonucleotide (ASO) and siRNA drugs [101]. Several clinical trials have demonstrated that ASO can effectively reduce LP(a) with a good safety profile. Studies are ongoing to determine whether selective reduction of LP(a) by ASO reduces major cardiovascular events [109]. The 2022 ACC Annual Meeting reported the results of Phase 1 clinical study of the APOLLO trial. In participants with Lp(a) ≥ 150 nmol/L and no known CVD, SLN360 (a siRNA) safely and effectively lowered Lp(a) levels in a dose-dependent manner [110]. Newer research indicates that olpasiran, another type of siRNA drug, can substantially decrease the levels of LP(a) in patients with ASCVD [111]. Results of follow-up trials on whether siRNA drugs can reduce adverse cardiovascular events are promising.

### Inflammation and residual cardiovascular risk

Burst analysis revealed that anti-inflammatory therapy is one of the research frontiers of residual cardiovascular risk. Inflammation constitutes a vital element in the pathophysiology of CVD. The disequilibrium amidst the pro- and anti-inflammatory mechanisms culminates in the chronic inflammation and the genesis of atherosclerotic plaques within the vascular wall. Although the degree of inflammation varies among individuals, specific biomarkers such as high-sensitivity C-reactive protein (hs-CRP) can serve as an indicator of residual ischemic risk [112]. In the last few decades, there has been a growing body of evidence demonstrating the significant role of inflammation in the development of coronary atherosclerosis. The CANTOS trial confirmed the role of anti-inflammatory treatment in reducing cardiovascular risk but also increased the risk of certain side effects, and currently, the FDA has not approved its use in patients with CAD [55, 113]. Conversely, the CIRT study found that low-dose methotrexate could not reduce cardiovascular events [92, 93]. Recently, the LoDoCo2 trial demonstrated that low-dose colchicine can safely reduce cardiovascular events, adding evidence to the potential of anti-inflammatory treatment for ASCVD. A large number of randomized controlled clinical trials targeting immunotherapy for atherosclerosis are currently underway [116].

### Dual-pathway inhibition

Aspirin is the standard treatment for atherosclerosis. Antiplatelet therapy is the cornerstone of preventing atherothrombotic events in patients with ASCVD. However, the advent of non-vitamin K antagonist oral anticoagulants (NOACs) agents has led to an emerging paradigm that is not limited to aspirin alone for the prevention of atherosclerotic thrombotic events. Dual-pathway inhibition (DPI) refers to the added use of an anticoagulant as an adjunct to antiplatelet therapy [117]. The efficacy and safety of DPI strategies have been the subject of most research interest. A meta-analysis showed that DPI therapy was effective in reducing ischemic events in patients with CVD compared with antiplatelet therapy alone, but with an increased risk of major and total bleeding [118]. Rivaroxaban 2.5 mg twice daily plus aspirin has been endorsed by guidelines as a secondary prevention strategy in patients with chronic coronary syndromes who are at moderate-to-high risk of ischemic events and not at high risk of bleeding [119]. The DPI strategy may be a viable approach to reduce residual cardiovascular risk. This approach is particularly important in certain patient groups, especially for those who have a higher risk of thrombosis due to heart disease or peripheral arterial disease (PAD) and who also require long-term antithrombotic therapy [120]. Future trials should more appropriately balance the patient's baseline ischemic risk with the expected increased risk of bleeding. Furthermore, additional research is imperative to ascertain the hazards and merits of alternative DPI tactics beyond rivaroxaban and aspirin.

Our research corroborates the significance of comprehensive lipid reduction in lowering the risk of cardiovascular events, echoing the findings of several recent studies that underscore the critical role of comprehensive lipid management in reducing residual cardiovascular risk [12, 121, 122]. With the advent of PCSK9 inhibitors and siRNA therapeutics, we have not only witnessed a diversification of treatment modalities but also observed how these novel therapies challenge and complement current treatment guidelines. In the future, combination therapy is expected to become more prevalent compared to monotherapy with statins [121]. Our data further support the notion of residual inflammation as a predictor of cardiovascular risk, aligning with the current research focus on residual inflammation as an independent risk factor for cardiovascular events [123]. Although current guidelines typically do not recommend anti-inflammatory medications for secondary cardiovascular prevention, the potential role of anti-inflammatory treatment in the management of atherosclerosis suggests that future clinical guidelines may need to incorporate anti-inflammatory strategies as a significant component of treatment. DPI therapy has been proven to offer significant benefits across various ASCVD populations and is cost-effective [124]. However, to ensure the widespread clinical application of DPI, precise assessment of bleeding and thrombotic risks in clinical research is essential to identify individuals who are most likely to benefit from this treatment. Furthermore, the exploration of new antithrombotic drugs with favorable safety profiles for implementing DPI should continue [125]. Future research on residual cardiovascular risk should place greater emphasis on collaboration at national and individual levels, encouraging partnerships between CVD researchers and experts in fields such as computer science, nutrition, and sociology to foster knowledge exchange and innovation in the field. Expanding research across different populations and regions and strengthening population-based cohort studies will ensure the universality of research findings. Utilizing the latest artificial intelligence and big data analytics technologies in disease prediction, diagnosis, and treatment can enhance the accuracy and efficiency of managing residual cardiovascular risk. Research outcomes should be better translated into policy recommendations, developing and implementing evidence-based public health strategies to reduce the overall burden of residual cardiovascular risk.

In order to validate our hypotheses and capture the latest developments in the field, we further reviewed the literature published from September 28, 2022, to November 21, 2023. As shown in Additional file 3: Figure 1, the research focus over the past year has continued to center on comprehensive lipid-lowering studies, with low-density lipoprotein remaining the core topic of discussion. The persistence of this trend further validates the basic accuracy of our analysis. Notably, as illustrated in Additional file 4: Figure 2, we observed that 'covid' and 'ckd' have emerged as new key terms. The appearance of 'covid' is likely related to the COVID-19 pandemic, but we speculate that it may not be the main direction for future research, as it does not occupy a central position in the keyword network. In contrast, 'ckd' (chronic kidney disease) is more central in the network and is associated with a greater number of research links. Particularly noteworthy is the concept of Cardiovascular-kidney-metabolic syndrome (CKM) introduced by AHA in October 2023, which positions kidney disease alongside heart and metabolic diseases in an integrated prevention and treatment framework [126]. In light of this, we predict that research on CKM will rapidly increase in the near future, thereby providing more comprehensive management strategies and evidential support for reducing residual cardiovascular risk.

### Limitations

This study applies bibliometric methods to analyze articles published over the past two decades, aiming to identify global trends and research hotspots in the field of residual cardiovascular risk. However, there are several limitations to this research. Firstly, our study relies on the WoSCC database and does not include other databases, which may result in the omission of some relevant studies. Secondly, we only considered articles and reviews, excluding conference papers and books, which could further contribute to publication bias. Lastly, due to the lag in literature dissemination, some recently published articles may not have accrued sufficient citations or widespread attention to be adequately represented in this study.

### Future directions

To address these limitations and enrich future research, the following measures are recommended: Expand the scope of research by integrating multiple databases to include a broader array of scholarly work. Incorporate a wider variety of document types to capture a more comprehensive global research perspective. Employ more advanced qualitative analysis methods to delve into the content quality of publications, reducing reliance on quantitative analysis and gaining a fuller understanding of the evolution of research contexts and theoretical frameworks.

## Conclusion

Based on the results of our bibliometric analysis, we draw the following conclusions: Research output related to residual cardiovascular risk has grown annually, reflecting sustained global scientific interest in this field. The USA has emerged as a leader and a hub for international collaboration. Significant contributions from prominent figures in the field have driven progress within the discipline. Leading publications have served as primary platforms for the dissemination of research, highlighting their central role in academic communication and the spread of information. Current research hotspots are focused on identifying and managing risk factors for residual cardiovascular risk, determining therapeutic targets, and developing corresponding pharmacotherapies. Comprehensive lipid reduction, residual inflammation risk assessment, and DPI strategies are not only research hotspots but also recent frontier trends, poised to become significant means of reducing residual cardiovascular risk in the future. In summary, our study supplements the bibliometric research on residual cardiovascular risk, analyzes the current state of research in this field, and summarizes the research hotspots and frontier trends.

### Supplementary Information

**Additional file 1.** Top 10 most cited journals in the field of residual cardiovascular risk.

**Additional file 2.** Top 10 articles by GC.

**Additional file 3.** Keywords co-occurrence network.

**Additional file 4.** Time-based of keyword co-occurrence network.

## Data Availability

The datasets used and/or analyzed during the current study are available from the corresponding author upon reasonable request.

## Abbreviations

WoSCC: Web of Science Core Collection
CVD: Cardiovascular disease
LDL-C: Low density lipoprotein cholesterol
BC: Betweenness centrality
PCSK9: Proprotein convertase subtilisin/kexin type 9
IF: Impact factor
JCR: Journal Citation Reports
NEJM: New England Journal of Medicine
JACC: Journal of the American College of Cardiology
EHJ: European Heart Journal
JAHA: Journal of the American Heart Association
CAD: Coronary artery disease
ACS: Acute coronary syndrome
HDL: High-density lipoprotein
HDL-C: High-density lipoprotein cholesterol
TG: Triglycerides
ApoB: Apolipoprotein b
EPA: Eicosapentaenoic acid
DHA: Docosahexaenoic acid
LP(a): Lipoprotein (a)
ASCVD: Atherosclerotic cardiovascular disease
GC: Global citations
LC: Local citations
R3i: Residual Risk Reduction Initiative
PROMINENT: Pemafibrate to Reduce Cardiovascular Outcomes by Reducing Triglycerides in Patients with Diabetes
AHA: American Heart Association
ACC: American College of Cardiology
ESC: European Society of Cardiology
EAS: European Atherosclerosis Society
CANTOS: Canakinumab Antiinflammatory Thrombosis Outcomes Study
COLCOT: Colchicine Cardiovascular Outcomes Trial
REDUCE-IT: Reduction of Cardiovascular Events with Icosapent Ethyl-Intervention Trial
ACI: Average citations per item
TLS: Total link strength
COURAGE: Clinical Outcomes Utilizing Revascularization and Aggressive Drug Evaluation
siRNA: Small interfering RNA
ASO: Antisense oligonucleotide
hs-CRP: High-sensitivity C-reactive protein
NOACs: Non-vitamin K antagonist oral anticoagulants
DPI: Dual-pathway inhibition
CKM: Cardiovascular-kidney-metabolic syndrome
