# Supplementary material for: Bibliometric analysis of residual cardiovascular risk: trends and frontiers
Source: J Health Popul Nutr. 2023 Nov 28;42:132. doi: 10.1186/s41043-023-00478-z (PMC10683255; doi:10.1186/s41043-023-00478-z)
Supplement: Supplementary file 1 — Additional file 1. Top 10 most cited journals in the field of residual cardiovascular risk. [file 41043_2023_478_MOESM1_ESM.docx]

**Supplementary Table 1 Top 10 most cited journals in the field of residual cardiovascular risk.**

| **Rank** | **Journals** | **JCR** | **IF** | **NP** | **TC** | **h-index** | |
| --- | --- | --- | --- | --- | --- | --- | --- |
| 1 | New England Journal of Medicine | Q1 | 176.079 | 2 | 2560 | | 2 |
| 2 | Journal of the American College of Cardiology | Q1 | 27.203 | 20 | 1757 | | 18 |
| 3 | Atherosclerosis | Q1 | 6.847 | 40 | 1554 | | 20 |
| 4 | European Heart Journal | Q1 | 35.855 | 19 | 1535 | | 16 |
| 5 | Circulation | Q1 | 39.918 | 14 | 1275 | | 13 |
| 6 | American Journal of Cardiology | Q3 | 3.133 | 32 | 1098 | | 17 |
| 7 | Lancet | Q1 | 202.731 | 3 | 852 | | 3 |
| 8 | Journal of Clinical Lipidology | Q1 | 5.365 | 21 | 833 | | 10 |
| 9 | Clinical Journal of the American Society of Nephrology | Q1 | 10.614 | 2 | 822 | | 2 |
| 10 | Current Atherosclerosis Reports | Q1 | 5.967 | 34 | 814 | | 12 |
