# Supplementary material for: Bibliometric analysis of residual cardiovascular risk: trends and frontiers
Source: J Health Popul Nutr. 2023 Nov 28;42:132. doi: 10.1186/s41043-023-00478-z (PMC10683255; doi:10.1186/s41043-023-00478-z)
Supplement: Supplementary file 2 — Additional file 2. Top 10 articles by GC. [file 41043_2023_478_MOESM2_ESM.docx]

**Supplementary Table 2 Top 10 articles by GC.**

| **Rank** | **Title** | **Journal** | **Author** | **Year** | **LC** | **GC** |
| --- | --- | --- | --- | --- | --- | --- |
| 1 | Niacin in patients with low HDL cholesterol levels receiving intensive statin therapy | NEW ENGL J MED | BODEN WE | 2011 | 153 | 1923 |
| 2 | Diabetic Kidney Disease: Challenges, Progress, and Possibilities | CLIN J AM SOC NEPHRO | ALICIC RZ | 2017 | 4 | 819 |
| 3 | Red blood cell transfusion: a clinical practice guideline from the AABB* | ANN INTERN MED | CARSON JL | 2012 | 4 | 705 |
| 4 | Bardoxolone methyl in type 2 diabetes and stage 4 chronic kidney disease | NEW ENGL J MED | DE ZEEUW D | 2013 | 6 | 637 |
| 5 | Inhibition of miR-33a/b in non-human primates raises plasma HDL and lowers VLDL triglycerides | NATURE | RAYNER KJ | 2011 | 5 | 547 |
| 6 | From C-Reactive Protein to Interleukin-6 to Interleukin-1: Moving Upstream To Identify Novel Targets for Atheroprotection | CIRC RES | RIDKER PM | 2016 | 20 | 467 |
| 7 | Biological markers of oxidative stress: Applications to cardiovascular research and practice | REDOX BIOL | HO E | 2013 | 0 | 461 |
| 8 | A Test in Context: Lipoprotein(a): Diagnosis, Prognosis, Controversies, and Emerging Therapies | J AM COLL CARDIOL | TSIMIKAS S | 2017 | 34 | 405 |
| 9 | Transfusion-related mortality: the ongoing risks of allogeneic blood transfusion and the available strategies for their prevention | BLOOD | VAMVAKAS EC | 2009 | 2 | 395 |
| 10 | Information provision for stroke survivors and their carers | LANCET | MAZZONE T | 2008 | 5 | 385 |
