# Supplementary figures and images for: Bibliometric analysis of residual cardiovascular risk: trends and frontiers
Source: J Health Popul Nutr. 2023 Nov 28;42:132. doi: 10.1186/s41043-023-00478-z (PMC10683255; doi:10.1186/s41043-023-00478-z)

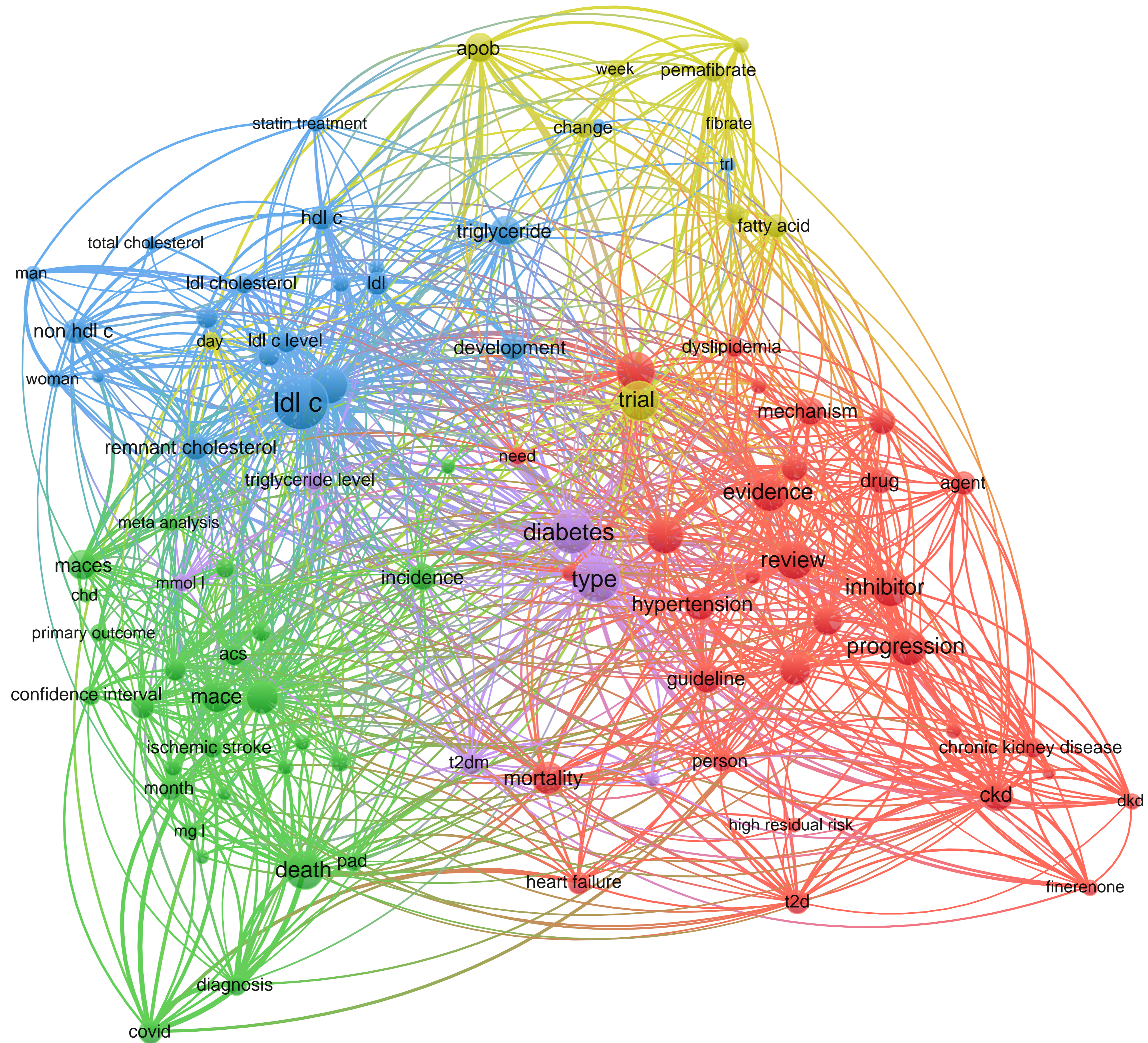

Supplement: Supplementary file 3 — Additional file 3. Keywords co-occurrence network. [file 41043_2023_478_MOESM3_ESM.pdf]
