# Supplementary material for: Bibliometric analysis of residual cardiovascular risk: trends and frontiers
Source: J Health Popul Nutr. 2023 Nov 28;42:132. doi: 10.1186/s41043-023-00478-z (PMC10683255; doi:10.1186/s41043-023-00478-z)
Supplement: Supplementary file 4 — Additional file 4. Time-based of keyword co-occurrence network. [file 41043_2023_478_MOESM4_ESM.pdf]

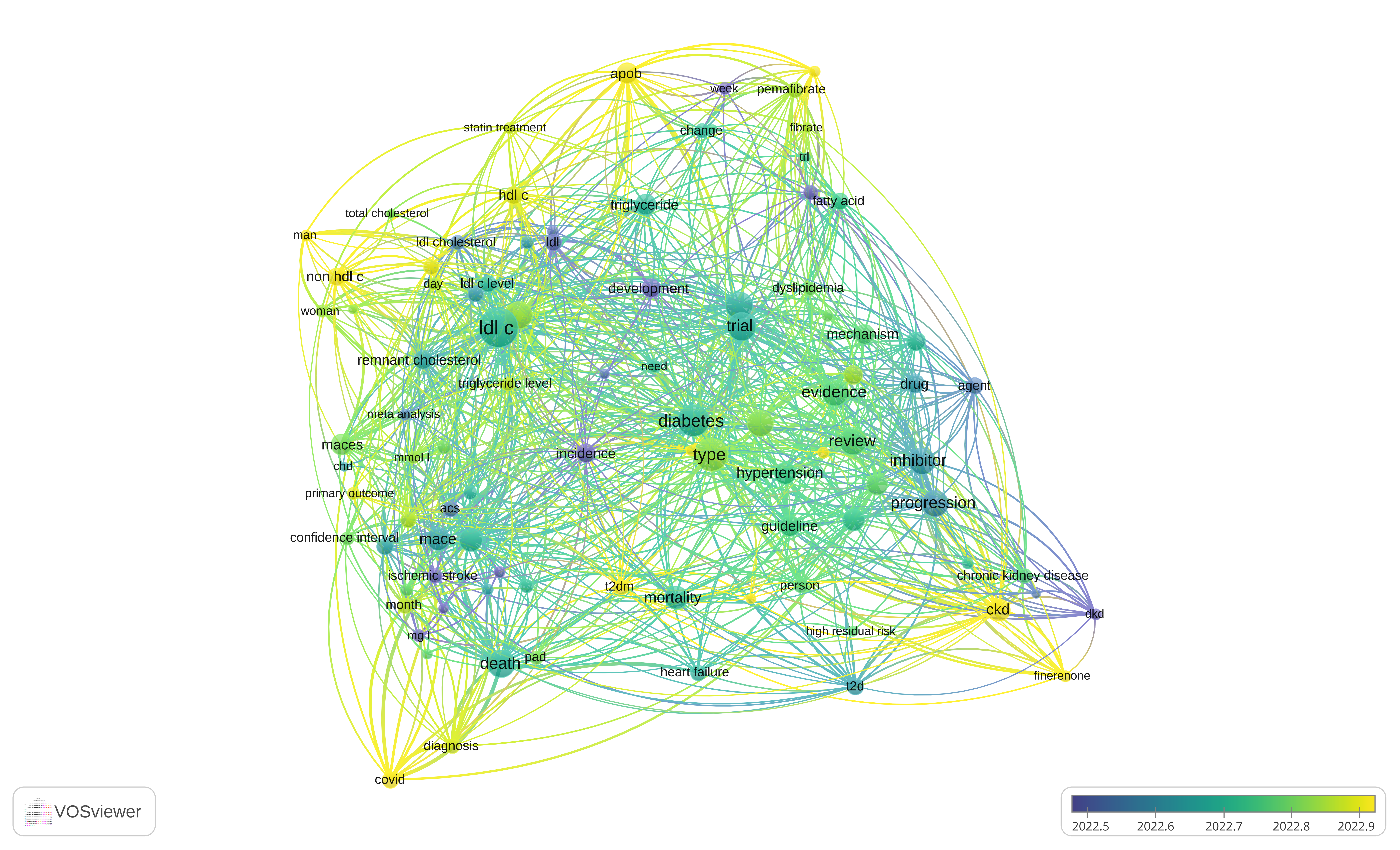

apob
week
pemafibrate
statin treatment
change
fibrate
trl
hdl c
triglyceride
fatty acid
total cholesterol
man
ldl cholesterol
ldl
non hdl c
day
ldl c level
development
dyslipidemia
woman
ldl c
trial
mechanism
remnant cholesterol
need
drug
agent
triglyceride level
evidence
meta analysis
diabetes
review
maces
incidence
type
inhibitor
mmol l
chd
hypertension
primary outcome
progression
acs
guideline
confidence interval
mace
ischemic stroke
chronic kidney disease
t2dm
person
mortality
month
ckd
dkd
high residual risk
mg l
pad
death
heart failure
finerenone
t2d
diagnosis
covid
VOSviewer
2022.5
2022.6
2022.7
2022.8
2022.9
